# From bench to policy: a critical analysis of models for evidence-informed policymaking in healthcare

**DOI:** 10.3389/fpubh.2024.1264315

**Published:** 2024-03-26

**Authors:** Seyyed Hadi Jabali, Shahram Yazdani, Hamid Pourasghari, Mohammadreza Maleki

**Affiliations:** ^1^School of Health Management and Information Sciences, Iran University of Medical Sciences, Tehran, Iran; ^2^Virtual School of Medical Education and Management, Shahid Beheshti University of Medical Sciences, Tehran, Iran

**Keywords:** evidence-informed policymaking, research utilization, knowledge translation, health policy, model, critical review

## Abstract

**Background:**

The use of research evidence in policy making is a complex and challenging process that has a long history in various fields, especially in healthcare. Different terms and concepts have been used to describe the relationship between research and policy, but they often lack clarity and consensus. To address this gap, several strategies and models have been proposed to facilitate evidence informed policy making and to identify the key factors and mechanisms involved. This study aims to critically review the existing models of evidence informed policy making (EIPM) in healthcare and to assess their strengths and limitations.

**Method:**

A systematic search and review conducted to identify and critically assess EIPM models in healthcare. We searched PubMed, Web of Science and Scopus databases as major electronic databases and applied predefined inclusion criteria to select the models. We also checked the citations of the included models to find other scholars’ perspectives. Each model was described and critiqued each model in detail and discussed their features and limitations.

**Result:**

Nine models of EIPM in healthcare were identified. While models had some strengths in comprehension, flexibility and theoretical foundations, analysis also identified limitations including: presupposing rational policymaking; lacking alternatives for time-sensitive situations; not capturing policy complexity; neglecting unintended effects; limited context considerations; inadequate complexity concepts; limited collaboration guidance; and unspecified evidence adaptations.

**Conclusion:**

The reviewed models provide useful frameworks for EIPM but need further improvement to address their limitations. Concepts from sociology of knowledge, change theory and complexity science can enrich the models. Future EIPM models should better account for the complexity of research-policy relationships and provide tailored strategies based on the policy context.

## Introduction

Evidence-informed policy making (EIPM) is a process that involves systematically and transparently finding, evaluating, and using the best available evidence as an input into decision making (1). The historical origin of EIPM can be traced back to before World War II, when governments invested public funds in scientific research with the expectation of various outcomes in military, economic, medical and other domains (2, 3). Evidence-based decision making in clinical care is another important precursor and parallel of EIPM. It emerged from the need to apply the growing and varied clinical research in a systematic and transparent way to improve patient outcomes and care quality (4). It also involves integrating the best research evidence with clinical expertise and patient preferences, and developing and implementing evidence-based guidelines and recommendations for clinical practice (5). The movement toward evidence-based decision making in clinical care has influenced the academic and policy-making communities to adopt a similar approach for health policy, as both domains share the common goal of improving health and well-being based on sound evidence (6).

However, the use of scientific evidence in policymaking has not been a smooth and linear process, but rather a turbulent and contested one. In the 1960s–1970s, UK and US policymakers experimented with improving research utilization (7, 8), spurring academic literature on the science-policy relationship and models of this linkage (8, 9). However, by the 1980s governments were questioning the value of research, especially social sciences, proposing budget cuts that highlighted consequences for academics with limited policy relevance (10). Despite these setbacks, the late 1990s saw a resurgence in using science for policymaking with the UK’s New Labor modernization agenda (11–13). Within public health, evidence-based medicine (14, 15) and WHO’s evidence-based health promotion (16) bolstered this. Consequently, much literature emerged on research-evidence and policy links (17), often following Weiss’s categorization of instrumental, conceptual and symbolic use (7). Still, even after four decades, researching the complexity of evidence use in policymaking remains challenging (18).

The relationship between research and policy can be described by different expressions, such as ‘evidence-informed policy making’, ‘evidence-based policy making’, ‘knowledge translation’, ‘knowledge transfer and exchange’, ‘knowledge to action’, ‘implementation science’, ‘research use in policy making’ etc. (19–23). These expressions may suggest different ways of understanding and applying research evidence in policy making (20). However, in reality, these expressions are often not clearly distinguished and their meanings are not well agreed upon (23–26). Despite the differences in terminology, the main ideas and principles of these expressions are similar. In this article, we use the term ‘EIPM’ as a general label for the various expressions that describe the relationship between research and policy. We define EIPM as using the best scientific evidence from various fields to inform public health policies and programs, with the aim of improving the quality and effectiveness of health outcomes. We acknowledge that research evidence is just one of several factors that influence policy decision making (27, 28) and that EIPM emphasizes the enhancement of scientific evidence use in policy making (29). We also recognize that other expressions may be used in the literature and practice to represent the same or similar concepts as EIPM, and we do not intend to exclude or disregard any of them in this article. Bridging the gap between research evidence and policy and practice in healthcare is an ongoing challenge (30–35). The relationship between research and policy is not a simple or rational one, but rather a messy, complex process that involves many factors and actors (36–38). Several strategies have been proposed to overcome this complexity and to increase the use of research among policymakers (39–41). These strategies mostly focus on increasing access to research, promoting interaction between research producers and users, and improving organizational capacity to use research (41).

However, despite these strategies to overcome barriers in public health, there is still a lack of literature on how to effectively promote and facilitate them (42). Models and frameworks provide a way to structure the existing knowledge in policy making and apply a more consistent method to select and test these strategies (43). In this field, there is no clear distinction between the terms ‘theories’, ‘models’ and ‘frameworks’, and they are often used synonymously (20, 44, 45). Many models, theories, and frameworks have emerged in the field of EIPM (21, 46). However, some critics have argued that there is insufficient guidance on how to choose the most suitable one (45). Many existing models have developed from an academic perspective, and are quite complicated in order to capture the complexity of the processes involved (47). As Powell et al. (48) reported that only a minority (4%) of the agencies that influence policy felt that most of the current frameworks for evidence-informed policy making were easy to implement. In this study, we aim to critically review the models of EIPM in healthcare and provide strengths and drawbacks of each one; we applied the following criteria to select the models for our review: originality, clarity, causality, comprehensiveness, level of policy making, and publication date. These criteria were chosen based on our research question and objectives, and were inspired by similar or related criteria used in previous studies (19, 49) that reviewed frameworks, interventions, or policies in different domains. The details of these criteria are explained in the method section.

## Method

A systematic search and review of the various models of EIPM in healthcare was performed in this study. This type of review is suitable for addressing broad questions and providing a best evidence synthesis, as it combines the strengths of a critical review with a comprehensive search process (50). Moreover, it aligns with our research objective, which is to explore the features and critiques of different models of EIPM in healthcare. This would help us to understand the diversity and complexity of the models. To achieve this, we applied the method described by Carnwell and Daly (51), who state that a critical review involves defining the scope of the review, identifying the source of relevant information, reviewing the literature, writing the review, and applying the literature to the proposed study.

### Defining the scope and identifying the source of relevant information

The scope of this review was to explore the features and critiques of the different models of EIPM in healthcare. We performed a systematic search of PubMed, Web of Science and Scopus databases from February 2023 to June 2023 using three categories of words; research dissemination or utilization, health policy or decision making, and framework or model. Only the models that satisfied the following criteria and provide practical guidance and support for evidence-informed policymaking were included in this review:

- Originality: The models included had unique or novel constructs and relations, compared to previous models, as defined by Shibayama and Wang (52) as bringing new perspectives to an existing body of knowledge.
- Clarity: The models included had clearly defined and operationalized constructs that were not ambiguous or broad, similar to the exclusion criteria used by Votruba et al. (19) in their systematic review of mental health evidence and policy frameworks.
- Causality: The models included that explained how their constructs influenced each other causally, semantically, procedurally or relationally.
- Comprehensive: The models included that covered all the stages of policy making, from agenda setting to evaluation, in a comprehensive manner.
- Level of policy making (53): The models included were either designed specifically for the legislative level of policy making based on this framework, or could be applied broadly to all three levels. We excluded models only suitable for the administrative or clinical levels, which would be narrower in scope. The level of policy making is categorized based on the framework proposed by Lomas (53) which describes three levels:
- Legislative: Refers to system-level policies created by elected officials at municipal, state/provincial, national or international levels.
- Administrative: Refers to organizational policies created by appointed managers and administrators in healthcare organizations.
- Clinical: Refers to policies created by healthcare professionals to guide clinical practice.
- Publication date: The models included that were published from the earliest available date in the literature.

For systematic search and review, quality assessment may or may not be included (50). We acknowledge that quality appraisal can be useful but we decided not to conduct a quality appraisal of the models that we included in our review because quality appraisal involves subjective judgments and interpretations that may vary depending on the perspective or context of the appraiser (54); and we wanted to avoid imposing our own views or values on the models. Moreover, we followed the examples of some authors who have conducted similar reviews of normative or theoretical literature, and who have justified their choice not to perform a quality appraisal (19, 54). Using title, abstract and full text, two reviewers (SHJ and SHY) screened the search results independently. Any disagreements were discussed and resolved by consensus between them. We recorded the number and reasons of studies included and excluded at each stage of the screening process.

### Reviewing the literature

**Figure 1:**
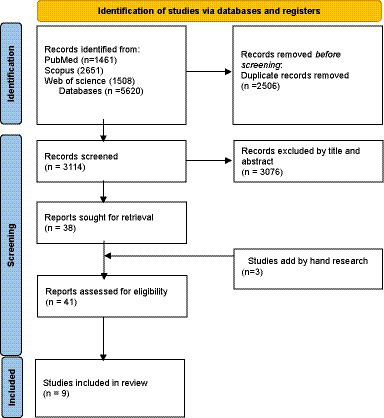
Study selection flow.

Through this process we identified the relevant sources of information for our review. We also checked all articles that cited the included models to find other scholars’ perspectives about the models. In the following sections, we will describe each model in detail, along with its original description and scholarly perspectives, and then we will discuss our own critique about different models of EIPM in healthcare. Figure 1 shows a PRISMA flow diagram of our selection process. Table 1 shows the reasons for including each model in our review.

**Table 1:** Reasons for including different models of evidence informed policy making.

Model name	Is it similar to previous models? If YES, which ones?	Year	Are its constructs well specified or are they very general and/or very vague?	Does it specify causal relations between its constructs?	Is it comprehensive in terms of policy making? If no what specific broad activities of policy making or research utilization are highlighted?	For which level of policy making is the model presented? (Legislative, administrative or clinical)	Selection
Ottawa Model of Research Use 1998 (55), 2004 (56)	NO	1998	Well specified	YES	YES	For all three level	Included
The PARIHS Framework 1998 (57), 2004 (58)	NO	1998	Well specified	YES	YES	Just clinical	Excluded
The RE-AIM Framework 1999 (59)	NO	1999	Well specified	NO	NO/Evaluation	Just clinical	Excluded
The Research Sequence (or Flow), the Knowledge Reservoir and the Assessment of Research Impacts 2000 (60), 2003 (61)	NO	2000	Well specified	YES	NO/Evaluation	For all three level	Excluded
EVIDENCE-BASED DECISION-MAKING 2000 (62)	NO	2000	Well specified	YES	YES	For all three level	Included
Stetler Model 2001 (63)	NO	2001	Well specified	YES	YES	Just clinical level	Excluded
Iowa model of evidence-based practice 2001 (64)	NO	2001	Well specified	YES	YES	Just clinical level	Excluded
Framework for Research Dissemination and Utilization 2002 (65)	NO	2002	Well specified	YES	YES	For all three level	Included
The Evidence-Informed Policy and Practice Pathway 2005 (27)	NO	2005	Well specified	YES	YES	For all three level	Included
The JBI model of evidence-based healthcare 2005 (66), 2022 (67)	NO	2005	Well specified	YES	YES	For all three level	Included
Theorical framework for the transformation of knowledge to policy actions 2006 (68)	NO	2006	Well specified	YES	YES	Just for legislative level	Included
Knowledge to action conceptual framework 2006 (25)	NO	2006	Well specified	YES	YES	For all three level	Included
Models for linking research to action 2006 (69)	NO	2006	Well specified	YES	YES	Just for legislative level	Included
Healthy public policy. Conceptual framework. 2007 (70)	NO	2007	Well specified	NO	NO/Implementation	Just for legislative level	Excluded
The Tehran University of Medical Sciences Knowledge Translation Cycle. 2008 (71)	YES, to 13,5,4	2008	Well specified	YES	YES	For all three level	Excluded
Participatory Action Knowledge Translation (PAKT) model 2008 (72)	YES 12	2008	Well specified	YES	YES	Just for clinical level	Excluded
Conceptual framework for healthcare decision making in a given setting. 2008 (73)	NO	2008	Well specified	YES	NO/Formulation	For all three level	Excluded
The Practical, Robust Implementation and Sustainability Model 2008 (74)	NO	2008	Well specified	YES	YES	Just for clinical level	Excluded
Translational framework for public health research. 2009 (75)	NO	2009	Well specified	YES	YES	Just for clinical level	Excluded
a consolidated framework for advancing implementation science 2009 (76)	NO	2009	It was vague	NO	NO/Evaluation	Just for clinical level	Excluded
Conceptual framework of the knowledge transfer process 2009 (77)	YES, to 8,12	2009	Well specified	YES	YES	For all three level	Excluded
Conceptual model of global factors affecting implementation in public service sectors 2011 (78)	NO	2011	Well specified	YES	NO/Implementation	Just for clinical level	Excluded
Framework for improving the use of evidence in managerial decision making 2012 (79)	NO	2012	Well specified	YES	YES	Just for administrative level	Excluded
Evidence-generating organizations in LMIC health systems. 2013 (80)	NO	2013	Well specified	NO	NO/Implementation	For legislative and administrative level	Excluded
Conceptual Model for Evidence-Informed Policy Formulation and Implementation 2015 (81)	YES, to 9	2015	Well specified	YES	YES	Just for legislative level	Excluded
The SPIRIT Action Framework. 2015 (43)	NO	2015	Wel specified	YES	YES	Just for legislative level	Included
Proposed Framework for Knowledge Translation in Ageing in Health 2017 (82)	YES, to 13	2017	Well specified	YES	YES	Just for legislative level	Excluded
The PHAST model for standardized public health data 2017 (83)	NO	2017	Well specified	YES	NO/Formulation and Implementation	Just for clinical	Excluded
The Context and Implementation of Complex Interventions (CICI) framework (84)	NO	2017	Well specified	YES	NO/ Implementation	For all three level	Excluded
FHI 360 Research Utilisation Framework 2018 (85)	YES, to 10	2018	Well specified	YES	YES	For all three level	Excluded
A model for increasing the use of evidence by decision-makers at multiple levels, raising their awareness, building capacity and supporting evidence use 2018 (86)	NO	2018	Well specified	YES	NO/Implementation	Just for legislative level	Excluded
Theoretical model on effective knowledge mobilization 2018 (87)	YES, to 2	2018	Well specified	YES	YES	Just for clinical level	Excluded
Evidence-based framework for evidence-based management in healthcare organizations 2018 (88)	NO	2018	Well specified	YES	YES	Just for administrative level	Excluded
The WHO-INTEGRATE 2019 (89)	NO	2019	It was general	YES	NO/Implementation and Evaluation	Just for clinical level	Excluded
EVITA framework 2019 (90), 2021 (91)	NO	2019	Well specified	YES	NO/Agenda setting	Just for legislative level	Excluded
Cynefin Framework for Evidence-Informed Clinical Reasoning and Decision-Making 2019 (92)	NO	2019	Wel specified	YES	NO/Formulation	Just for clinical level	Excluded
Working conceptual model for embedded implementation research. 2020 (93)	NO	2020	Well specified	YES	NO/Evaluation	Just for legislative level	Excluded
IWH Research Impact Model 2020 (94)	YES, to 13,4	2020	Well specified	YES	NO/Evaluation	For all three level	Excluded
synthesized framework of evidence-based decision-making in health system management 2022 (95)	YES, to 10	2022	Well specified	YES	YES	Just for administrative level	Excluded
SMILE framework 2022 (96)	NO	2022	Well specified	YES	NO/Agenda setting	For all three level	Excluded
A comprehensive monitoring and evaluation framework for evidence to policy networks 2022 (97)	NO	2022	Well specified	YES	NO/Evaluation	Just for legislative level	Excluded

## Result

We followed the Preferred Reporting Items for Systematic reviews and Meta-Analyses (PRISMA) statement and used the PRISMA 2020 flow diagram for new systematic reviews (98) which included searches of databases, registers and other sources to report the study selection process, even though our paper is not a systematic review, but a systematic search and review. We retrieved 5,620 records from the databases and excluded 3,076 records after screening their titles and abstracts. We obtained the full texts of the remaining 38 records and assessed them for eligibility. We also added two records by hand searching. A PRISMA flow diagram was used to depict the study selection process (Figure 1), as it provides a clear way to report the screening and inclusion of studies for a systematic search. We excluded 31 records that did not meet the inclusion criteria. We included nine studies in our review. They consist of nine models of evidence-informed policy making. These models and framework are well-known and widely used in the field of health policy and systems research. We will provide a detailed description and analysis of each of them in the following sections.

1. The Ottawa model of research use (OMRU)
2. The Canadian Health Service Research Foundation model (CHSRF)
3. The Framework for Research Dissemination and Utilization (FRDU)
4. The Evidence-Informed Policy and Practice Pathway (EIPPP)
5. The JBI model of evidence-based healthcare (JBI)
6. Theoretical framework for the transformation of knowledge to policy actions (TKPA)
7. Knowledge to action process model (KTA)
8. Models for linking research to action (MLRA)
9. The SPIRIT Action Framework (SPIRIT)

### Ottawa model of research use (OMRU)

According to Logan and Graham, the developers of the OMRU, this six-step framework provides a valuable approach for implementing and evaluating healthcare innovations in the context of evidence-informed policy making. The OMRU offers a comprehensive and dynamic approach to knowledge transfer, incorporating elements such as evidence-based innovation, potential adopters, and implementation interventions. The model emphasizes the importance of assessing, monitoring, and evaluating each element to overcome barriers and enhance supports for successful knowledge transfer. The six steps involve identifying individuals with the authority to implement changes, clarifying the details of the innovation, assessing barriers and supports to adoption, planning and tailoring implementation interventions, measuring and monitoring adoption, and evaluating the impact of the innovation on health, practitioner, and system outcomes (55, 56, 99). Analyzing the strengths and limitations of the OMRU provides insights into its utility as a model for evidence-informed policymaking, which aligns with the central aim of this review in assessing models that facilitate evidence-informed policymaking.

The OMRU and its strengths and limitations have been discussed by various authors. The OMRU is praised for its operational focus, which outlines the actions for effective implementation of behavior change interventions (100), its dynamic and interactive approach to research and knowledge translation, which involves stakeholders and reflects complex systems thinking (101, 102), its assistance to administrators to control factors influencing organizational-level changes (103), and its usefulness to facilitate knowledge translation at the local context, especially for continuity of care interventions that bridge sectors, settings and provider groups (104). The OMRU also follows the principle that knowledge translation strategies should be tailored to the specific barriers and supports of each setting, making it efficient and adaptable (105), and it is clear and easy to use (106). Moreover, the OMRU is one of the few frameworks that address implementation at multiple socio-ecological levels (107). However, the OMRU also has some limitations, such as the high demand for time and resources, the lack of specific guidance on how to choose and apply knowledge translation strategies, and the potential challenges for its applicability in low-resource setting (103). The OMRU also focuses on the clinical practice setting rather than the health system as a whole, needs further development and validation of tools and instruments to support its implementation, and does not cover research production or knowledge creation as part of knowledge translation (106). Additionally, the OMRU may limit the scope of the investigation and overlook some factors that are not included in the framework (108). According to the original authors of the model, some of the strengths of the OMRU are that it is flexible, interactive, and practical, while some of the weaknesses are that it is complex, time-consuming, and challenging (99). However, these perceptions may vary depending on the users and the contexts of the OMRU’s application. Some users of the OMRU have reported that it is clear and easy to use (106), while others have found it difficult and demanding (103). Therefore, the OMRU’s usability may depend on the specific situation and the needs of the users.

### The Canadian Health Service Research Foundation model (CHSRF)

The CHSRF model was first published in 2000 by the Canadian Health Services Research Foundation (CHSRF), which is now called the Canadian Foundation for Healthcare Improvement (CFHI), to address the gap between research and practice in the health sector (109). It is also known as the Lomas model, after Jonathan Lomas, the first chief executive officer of the CHSRF, who led the development of the model (109). The model suggests creating communication channels among four key groups, namely researchers, decision makers, research funders and knowledge purveyor. It also emphasizes the need to enhance the ability and willingness of decision makers and their organizations to access and apply research evidence. It acknowledges that different kinds of decision makers face various uncertainties about facts and values, and that research findings are often not useful or applicable for their needs. It aims to promote health-related research, especially in the areas of pharmaceuticals, technology and medicine-related innovations. It shows the diversity of actors in each category and how they provide information and resources to each other (62).

The CHSRF model has been challenged by various authors who identified its advantages and disadvantages. Evaluating the pros and cons of the CHSRF model contributes to the overall goal of this review in assessing models that aim to facilitate the use of research evidence in policy decisions. The model has some advantages, such as its focus on linkage and exchange, which fosters trust and mutual exchange of knowledge and influence between researchers and decision makers (110), its consistency with the approach of Weiss (8) and Caplan (9), who conceptualized evidence-based policy making as an interplay between a supply of and demand for evidence (86), its attention to improving relationships and communication across the four groups in the health sector (62), and its relevance for understanding and influencing policy communities, funding and brokering aspects of research-policy nexus (111), and its promotion of interaction between the producers and users of research, which is a necessary strategy for health researchers in Canada (112). However, the model also has some challenges, such as its reliance on complex and variable partnerships between academics and non-academic partners, which could cause conflicts or misunderstandings (113), and its lack of development in the research literature, which may limit its theoretical and empirical foundations (112).

### The Framework for Research Dissemination and Utilization (FRDU)

One of the comprehensive models that aim to support policymakers in using research evidence in their decision-making is the FRDU, which was developed by Dobbins et al. (65). This framework draws on the diffusion of innovations theory and integrates the concepts of research dissemination, evidence-based decision-making and research utilization. It suggests that four sets of characteristics influence the innovation adoption process for policymakers who want to adopt research evidence into their policies. These characteristics are innovation-related, individual, organizational and environmental. The framework also outlines the five stages of innovation adoption, which are knowledge, persuasion, decision, implementation and confirmation. The framework synthesizes relevant literature from the management and health-care fields, and is supported by empirical data from various studies. The original authors of the framework evaluated its strengths and weaknesses, as well as its challenges and facilitators for implementation. Analyzing the strengths and weaknesses of the FRDU framework provides insights into its utility for evidence-informed policymaking, connecting to the main aim of this review. They found that the framework was clear, applicable and useful, but also simple, linear and context-free. They also identified the scarcity of resources as the main challenge to implementing the framework, and having a champion or an external factor as the main facilitator. The authors of the framework intend to continue testing and refining it based on new knowledge and feedback.

FRDU has both strengths and weaknesses, as various authors have reported. Some of the strengths are that it considers constructs and stakeholders at different levels of the health care system (114), it helps to clarify the stages, areas, complexities, and variables of research dissemination and utilization process and evidence-informed decision-making in health care field and policy practices (115), and it has a strong theoretical and conceptual basis that captures the complexity and diversity of evidence-informed decision making and supports the role of communities of practice in facilitating the use of research evidence (116). However, the framework has some limitations and gaps. It suggests a one-way and limited relationship between researchers and knowledge users, which may not capture the complexity and reciprocity of evidence-informed decision-making (114). The framework’s reliability and usefulness are questionable due to the lack of empirical support or validation in real-world settings, which calls for more research to provide more evidence for the framework (117). Another drawback is that the framework does not have a specific or comprehensive theoretical foundation for specific knowledge translation mechanisms, such as evidence briefs (118). Moreover, the framework ignores the political and dynamic context of policy making, especially in low-income countries, which may affect the use of research evidence (119).

### The Evidence-Informed Policy and Practice Pathway (EIPPP)

This model, developed by Bowen and Zwi (27), proposes a three-stage process for how evidence can inform policy and practice in different contexts. The first stage is sourcing the evidence, which involves finding and selecting various types of evidence that are relevant and responsive to the policy and practice issue. The second stage is using the evidence, which involves applying and interpreting the evidence in the policy and practice context. The third stage is implementing the evidence, which involves enacting and evaluating the evidence-based innovation. The model also considers the policy context, the decision-making factors, and the capacity for implementation as key influences on each stage of the process. The model draws on literature from health, public policy, social sciences, and capacity building to provide a comprehensive and dynamic framework for evidence-informed policy and practice.

The EIPPP has much to offer, but it also has some limitations that need to be considered. The model has several advantages, such as suggesting that policy is largely shaped by evidence and can respond to the community needs (120), connecting evidence-based policy-making with capacity building and enabling context-appropriate policies (121), focusing on three socio-ecological levels in the decision-making process, emphasizing the policy context and its influence on the interaction between research, evidence, and the policy process (122), identifying the key points of intervention in the pathways to evidence-informed policy and practice, and taking into account the contextual and decision-making factors and the policy influences (123), being applied to inform an intervention design and explaining the significance of policy processes and evidence use in local government planning (124), assisting researchers and policy actors in navigating the use of evidence (125), complementing other theories of knowledge utilization by highlighting how external factors affect the use of evidence in decision making (126), and showing how evidence may be used in the decision-making process (123). However, the model also has some limitations that need to be considered, such as not linking capacity building and interactive ‘knowledge to action’ in a consistent and theory-based way (121), focusing on evidence, how it is spread and applied with little attention to the political and changing policy-making context in low income countries (119), not accounting for knowledge generation beyond a policy idea (123), and being limited in the detail given to the influences on decision making (123). Evaluating the advantages and drawbacks of the EIPPP contributes to the central objective of this article in assessing different models for their utility in facilitating evidence-informed policymaking.

### The JBI model of evidence-based healthcare (JBI)

The JBI is a model that guides how evidence is generated, synthesized, transferred and implemented in healthcare settings. It was developed by the Joanna Briggs Institute (JBI), an international research organization and collaborative network based in the University of Adelaide, South Australia (66). The JBI Model was updated in 2019 (127) to reflect the changing international discourse relating to evidence and its translation into health policy and practice. The JBI Model proposes overarching principles related to culture, capacity, communication, and collaboration (the 4 C’S) and considers different types of evidence that address the feasibility, appropriateness, meaningfulness and effectiveness (FAME) of healthcare practices. The JBI Model consists of five components that are evidence generation, evidence synthesis, evidence transfer, evidence implementation and global health. Evidence generation is about asking relevant and meaningful questions that reflect the needs and concerns of health professionals and patients. Evidence synthesis is the process of combining and summarizing different types of evidence to inform healthcare decision making. Evidence transfer is the process of sharing and integrating evidence into healthcare practice and policy. Evidence implementation is the process of applying evidence into healthcare practice and policy. Global health is the component that represents both the global and local aspects of evidence-based healthcare. The JBI Model emphasizes the critical relationship between research and practice and policy and the importance of stakeholder engagement, the localization of knowledge, responsiveness to local knowledge needs, shared decision-making and sustainability (67).

The JBI model is praised for being a comprehensive and inclusive model that integrates and accommodates different components and kinds of evidence in healthcare (128). It provides insights into how to apply and use knowledge in diverse and complex healthcare settings around the world (128). Moreover, the model incorporates various aspects of healthcare, such as needs, research production, synthesis, transfer and implementation, and takes into account the variety of contexts and evidence types that are involved in evidence-based healthcare (129). Analyzing the strengths of the JBI model aligns with the overall goal of this review in evaluating the utility of different frameworks for evidence-informed policymaking.

### Theoretical framework for the transformation of knowledge to policy actions (TKPA)

Ashford et al. (68) developed TKPA to guide how knowledge can be translated into health policy-making. The framework consists of three key activities that can create a window of opportunity for policy change that are agenda-setting, coalition building, and policy learning. Agenda-setting is the activity of defining and prioritizing the problems and solutions that need policy attention. Coalition building is the activity of forming and maintaining alliances and networks among actors who have a common interest or goal in influencing policy. Policy learning is the activity of acquiring and applying knowledge and skills to inform policy decisions. The framework was also applied in Kenya to incorporate evidence from a national assessment of health services into decentralized planning at the district level. The application of the model revealed some lessons and challenges, such as the importance of engaging stakeholders at all levels, the need for capacity building and technical support for data collection, analysis and reporting, the challenge of ensuring data quality, availability and accessibility, and the need for a clear policy, legal and institutional framework to guide public participation and inter-governmental coordination.

Robertson (130), praises TKPA for adding a new element of policy learning, which emphasizes the importance of using data and information to influence policy decisions. He also gives an example of how Ashford showed that scientific evidence and research results helped create health plans that were accepted by the Kenyan government. He suggests that Ashford’s model is better than Kingdon’s policy window, because it has a new component of policy learning. Similarly, Votruba et al. (19) describe the TKPA as an effective and appealing approach to create opportunities for policy change in Kenya, as it involves action through three key elements: setting the agenda, building coalitions and learning from data and information. Reviewing the TKPA model contributes to the central aim of this study in assessing frameworks that facilitate the use of research evidence in policy decisions.

### Knowledge to action process model (KTA)

One of the models that aims to explain and guide the process of moving knowledge into action is the knowledge to action framework, developed by Graham et al. (25). The framework is based on a concept analysis of 31 planned action theories and covers both the aspects of knowledge creation and action. Knowledge creation involves the generation, synthesis, and adaptation of knowledge to different contexts and audiences. It also involves three types of knowledge, which are knowledge inquiry, knowledge synthesis, and knowledge tools/products. Knowledge inquiry refers to primary research that produces new evidence. Knowledge synthesis refers to systematic reviews that summarize and integrate existing evidence. Knowledge tools/products refer to guidelines, decision aids, or other tools that present knowledge in a clear and usable way. Action involves the identification of a problem or gap, the selection and tailoring of knowledge, the implementation and evaluation of interventions, and the monitoring and sustainability of outcomes. It also involves seven phases of action, which are identify problem, identify, review, and select knowledge, adapt knowledge to local context, assess barriers and facilitators to knowledge use, select, tailor, and implement interventions, monitor knowledge use, and evaluate outcomes. The framework emphasizes the importance of collaboration, communication, and context in the process of knowledge translation. It also recognizes that knowledge creation and action are dynamic and iterative processes that require feedback loops and ongoing evaluation. The framework aims to bridge the gap between research and practice by providing a comprehensive and practical guide for knowledge translation.

The knowledge to action process model is widely used and recognized for its many advantages in evidence-informed policy making (131–133). It provides a clear and comprehensive framework for understanding and facilitating knowledge translation and integrated knowledge translation (134), with a unique theoretical foundation grounded in social constructivism (133). The model offers direction on key elements to consider in the knowledge translation process, such as barriers, facilitators, stakeholders, and context (135), as well as a general and flexible guide for implementation that allows for adaptation to different situations (134). With a strong evidence base and validity (131), the model can support the process of translating research evidence into practice by providing a systematic framework and various methods to overcome barriers, measure outcomes, and ensure sustainability (24). The model is a dynamic and interactive process that involves creating and improving knowledge to make it more relevant and applicable for different knowledge users, such as health professionals and policy makers (136). Additionally, the model provides a detailed picture of the actions and strategies that can be undertaken for research use in practice (137). However, despite its strengths, the model does have some limitations. For example, it does not specify how to measure the success of integrating research evidence into policy and practice (138) or offer detailed guidance on how to perform each step of the action cycle. Its suitability as an account of the knowledge transfer process is also largely unknown (77) and it may not be applicable or relevant in low-income countries where the policy making process is influenced by various factors beyond evidence (119). Additionally, the model lacks details on the knowledge synthesis phase (137) and does not link the knowledge creation and action phases well (137). Analyzing the advantages and disadvantages of the KTA model connects back to the overall objective of this review in evaluating different frameworks for their utility in evidence-informed policymaking.

### Models for linking research to action (MLRA)

An additional model of evidence informed policy making is MLRA, which was developed by Lavis et al. (69) to assess country-level efforts to use research for health policy and systems decisions. The model consists of four elements and four clusters of activities. The four elements are (1) the general climate for research use, which reflects the extent to which research funders, universities, researchers and users value and support the linkage between research and action; (2) the production of research, which involves identifying users’ needs, conducting scoping reviews, systematic reviews and single studies to address these needs, and synthesizing the research findings for users; (3) the evaluation of efforts to link research to action, which involves measuring the processes and outcomes of the linkage activities and using the results for improvement; and (4) the mix of clusters of activities used to link research to action. The four clusters of activities are (A) push efforts by producers or purveyors, which are strategies to disseminate research messages to potential users; (B) user pull efforts, which are strategies to enhance users’ capacity and motivation to use research evidence; (C) exchange efforts, which are strategies to foster partnerships and collaborations between researchers and users; and (D) integrated efforts, which are strategies that combine elements of push, pull and exchange efforts. The model provides a comprehensive framework for understanding and enhancing the use of research evidence in policy making.

While the model for linking research to action has been widely adopted and accepted in the health policy arena as a useful tool for understanding and improving the use of research evidence in policy making (139), it is not without its limitations. Despite its many strengths, such as providing detailed guidance on research dissemination activities (137) and accounting for various factors that affect research-policy interactions (137), the model has some shortcomings. For example, it does not specify the most influential factor for research dissemination and use (137) and neglects the design stage of research and other actors besides policy makers (140). Additionally, the model does not make clear how health information is valued or utilized by different policy actors or stakeholders and does not consider contextual challenges or opportunities that may affect the adoption or implementation of research evidence in different settings or situations (141). The model also does not address how funding agencies set their research priorities or respond to the needs and demands of policy makers and other stakeholders (142). Furthermore, the model is partially based on research evidence that has not been evaluated or refined, which may affect its validity, reliability, and relevance for different contexts and situations (143). Critically reviewing the MLRA model contributes to the main goal of assessing frameworks that aim to facilitate the use of research evidence in policy decisions.

### The SPIRIT action framework (SPIRIT)

A recent model of evidence informed policy making that guides the selection and testing of strategies to increase the use of research in health policy is the SPIRIT Action Framework (43). The authors claim that it guides the selection and testing of strategies to increase the use of research in health policy. The model has four main elements, catalysts, capacity, research engagement actions, and research use. Catalysts are events or situations that trigger the need or opportunity for using research in policy. Capacity is the ability of the policy organization and its staff to value, access, appraise, generate and interact with research. Research engagement actions are the activities that policy makers do to engage with research and apply it to their work. Research use is the outcome of applying research to inform policy making. Research use can be classified into four types: instrumental, conceptual, tactical, and imposed. Research use can occur at different stages of policy making: agenda setting, policy development, policy implementation, and policy evaluation. The model also recognizes that policy influences affect all elements of the framework. Policy influences are external factors that shape the policy context and the demand for research. They include public opinion, media, economic climate, legislative policy infrastructure, political ideology and priorities, expert advice, stakeholder interests, resources, and research.

The SPIRIT Action Framework has several notable strengths, but it also faces some challenges and limitations. One of the strengths of the framework is that it provides a clear and practical guidance for studying the causal pathways between research and policy and testing the effectiveness of different strategies to increase research use in policy (144). Another strength of the framework is that it has a clear aim, a sound knowledge base, a practical guidance, and a knowledge-building potential for improving research use in policy and practice (106). Some of the other strengths of the framework are that it covers the agenda-setting stage, recognizes the diverse roles of research in policy, and provides practical strategies and tools for implementation, such as SEER, ORACLE and SAGE (19). Moreover, it is based on a rigorous and systematic trial of different interventions and measures, which has refined and validated the framework and its tools based on empirical evidence and feedback (145). Furthermore, it comprehensively and realistically depicts the various influences on policy decisions, such as the actors, context, and structure, and how they affect the use of research in policy (146). On the other hand, one of the challenges and limitations of the framework is that it is still under evaluation and lacks published evidence to support its validity and effectiveness. The framework may need further improvement and may face difficulties in being widely used by policy makers and researchers who may prefer more proven frameworks (147). Another limitation of the framework is that it ignores the role of stakeholders in EIPM and only focuses on the use of research evidence in policy (148). Evaluating the strengths and limitations of the SPIRIT Action Framework provides insights into its utility for evidence-informed policymaking, connecting back to the central thesis of analyzing EIPM models in this review.

### The models of EIPM under the microscope: our findings

In light of the detailed description of the all nine models of evidence informed policy making provided above, we will critique all nine models of evidence informed policy making, as well as their implications for policy making practice in this section, from our own perspective. We concur with the existing critiques of these models, but we also recognize their value in providing a framework for using research evidence in policy decisions. Therefore, we also propose that these models need further improvement and adaptation to address their shortcomings.

One of the main limitations of most models, except for FRDU, MLRA and SPIRIT action framework, is that they assume that policy making is a rational process that is informed by research evidence as *a priori*. However, this assumption may not reflect the reality of policy making in many contexts, especially in low-and middle-income countries (LMICs) where decisions are often made in a centralized and non-transparent manner. Therefore, these models may not be suitable or applicable for such contexts.

Another limitation is that only two models (FRDU, and MLRA) explicitly mention the importance of being aware of the general atmosphere of the policy context, but they do not explain how to act accordingly. Similarly, SPIRIT action framework introduces the concept of “catalyst” as events or situations that trigger the need or opportunity for using research in policy, but it does not provide guidance on how to identify or respond to such catalysts. These models do not address the challenges of time-sensitive or crisis situations, such as the COVID-19 pandemic or the emergence of Ebola, where policy makers may face uncertainty, urgency and pressure to act quickly.

A third limitation is that none of the models consider the complexity of the problem and the approach that is required when dealing with wicked issues. Wicked issues are problems that are difficult to define, have multiple causes and effects, involve conflicting values and interests, and have no clear or definitive solutions (149). Examples of wicked issues in healthcare include obesity, mental health and aging. These issues require a different type of EIPM that is more adaptive, participatory and systemic (150).

A fourth limitation is that most models overlook the role of cognitive shortcuts and social norms in policy making. Cognitive shortcuts are mental strategies that people use to simplify complex information and make decisions quickly and efficiently (151). Social norms are unwritten rules that govern the behavior and expectations of individuals within a group or society (152). Both cognitive shortcuts and social norms can influence how policy makers perceive, interpret and use research evidence in their decisions. For example, policy makers may rely on heuristics (such as availability, representativeness or anchoring) to judge the relevance, quality or credibility of research evidence. They may also conform to the opinions or preferences of their peers, superiors or constituents, regardless of what the evidence says. Only one model (OMRU) implicitly considers these factors in one of its elements (“concern”), but it does not explain them explicitly or provide strategies to address them.

A fifth limitation is that none of the models provide an explicit explanation about the proportion of external factors such as values in the provided solution or policy options, or how much adaptation of research evidence is acceptable or desirable. All models acknowledge that research evidence is not the only factor that influences policy decisions, and that other factors such as values, interests, resources and feasibility also play a role. Some models use different terms to refer to the combination of research evidence and external factors, such as innovation (OMRU and FRDU), policy idea (CHSRF and EIPPP) or adapted version of research evidence (The JBI model, TKPA, MLRA and the SPIRIT action framework). However, none of them specify how much weight should be given to each factor, or how much modification of research evidence is allowed or recommended to make it more adoptable.

A sixth limitation is that all models emphasize the importance of collaboration between researchers and policy makers or engagement of stakeholders in EIPM, but none of them consider the heterogeneity of these groups or provide clear guidance on how to facilitate this collaboration. Researchers and policy makers have different backgrounds, knowledge, experiences and responsibilities, which may create challenges for communication, trust and mutual understanding. Stakeholders may also have diverse and conflicting interests, values and perspectives, which may affect their willingness and ability to participate in EIPM. Therefore, it is not enough to simply state that collaboration or engagement is necessary, but also to explain how it should be done, what strategies should be used, and what outcomes should be expected.

Despite these limitations, the models also have some strengths that are worth noting. For example, OMRU accounts for the user’s perception of innovation or policy idea, which is an important factor for adoption and implementation. The CHSRF Model recognizes the role of research funders as key actors in EIPM, who can influence the supply and demand of research evidence. Three models (TKPA; SPIRIT and EIPPP) incorporate a dynamic view of change theory in their models, which acknowledges that EIPM is influenced change theory. Also, KTA recommends that the knowledge creation and dissemination should be adjusted to the user needs, which is consistent with the principles of knowledge translation. A summary of the strengths and drawbacks of the models, based on both previous and our own critiques, is presented in Table 2.

**Table 2:** Summary of strengths and limitations of different models of evidence-informed policy making in healthcare based on existing critiques and our own analysis.

Included models	Other scholars’ critique		Our critiques	
	Strengths	Drawbacks	Strengths	Drawbacks
**OMRU**	Operational focus (100). Dynamic and interactive approach (101, 102). Stakeholder involvement (101). Complex systems thinking (102). Local context facilitation and Continuity of care interventions (104). Tailored knowledge translation strategies (105). Efficient and adaptable approach (105). Clear and easy to use (106). Multiple socio-ecological levels (107).	High demand for time and resources (103). Lack of specific guidance (103). Limited applicability in low-resource contexts (103). Narrow focus on clinical practice setting (106). Need for further development and validation (106). Omission of research production or knowledge creation (106). Possible scope limitation and factor omission (108).	Emphasizing innovation or idea over evidence. Accounts for the users’ perception of the innovation.	Presupposes a rational approach to policy-making. Lacks alternatives for time-sensitive or crisis situations. Not capture the diversity and complexity of policy problems and contexts. Assumes availability and quality of evidence. Neglects the unintended or unexpected effects of policy actions. Detail and specificity on policy maker-researcher relationship are missing.
**CHSRF**	Linkage and exchange (110). Consistency with evidence-based policy making (86). Relationship and communication improvement (62). Relevance for policy communities, funding and brokering (111). Interaction between producers and users of (112).	Complex and variable partnerships (113). Lack of development in the research literature (112).	Emphasizing idea over evidence. Distinguishing four types of actors for evidence-informed decision making. Recognizing the role of research funders as a key group.	Presupposes a rational approach to policy-making. Lacks alternatives for time-sensitive or crisis situations. Not capture the diversity and complexity of policy problems and contexts. Assumes availability and quality of evidence. Neglects the unintended or unexpected effects of policy actions. Overlooks cognitive shortcuts and social norms in policy making. Does not consider organizational change theories. Does not clarify how the communication should happen.
**FRDU**	Multiple constructs and stakeholders addressed (114). Stages and variables of research dissemination and utilization clarified (115). Strong and comprehensive theoretical basis (116). Communities of practice supported (116).	One-way and limited relationship assumed (114). Lack of empirical support or validation (117). No specific or comprehensive theoretical basis for knowledge translation (118). Political and dynamic context of policy making ignored (119).	Emphasizing innovation or idea over evidence.	Presupposes a rational approach to policy-making. Lacks alternatives for time-sensitive or crisis situations. Not capture the diversity and complexity of policy problems and contexts. Assumes availability and quality of evidence. Neglects the unintended or unexpected effects of policy actions. Overlooks cognitive shortcuts and social norms in policy making. Omits the role of knowledge brokers and purveyors.
**EIPPP**	Evidence-policy-community alignment (120). Evidence-policy-capacity-context linkage (121). Three socio-ecological levels, policy context and interaction addressed (122).	Lack of consistent and theory-based link (121). Political and changing policy-making context neglected (119).	Emphasizing innovation or idea over evidence.	Presupposes a rational approach to policy-making. Lacks alternatives for time-sensitive or crisis situations. Not capture the diversity and complexity of policy problems and contexts. Neglects the unintended or unexpected effects of policy actions. Overlooks cognitive shortcuts and social norms in policy making.
	Key intervention points, factors and influences pinpointed (123). Intervention design, policy processes and evidence use informed (124). Researchers and policy actors guided in using evidence (125). Other knowledge utilization theories enhanced by external factors’ impact (126). Evidence use in decision making demonstrated (123).	Knowledge generation beyond policy idea ignored (123). Limited detail on decision making influences (123).	Incorporates a complex and dynamic view of change theory.	Does not explicitly recognize the role of evidence in agenda setting. Overlooks power dynamics or political interests in evidence use and implementation. Assumes homogeneity between policy makers and researchers. Ignores conflicts or trade-offs between evidence types or sources. Omits the role of knowledge brokers and purveyors. Lacks guidance or strategies on assessing, developing, or enhancing capacities.
**JBI**	Comprehensive and inclusive model with diverse evidence integration (128). Insights into knowledge application and use in various and complex settings (128). Coverage of different aspects of healthcare and diversity of contexts and evidence types (129).		Incorporates evidence summaries as a streamlined and timely approach to evidence synthesis. Integrates the expertise of policy makers and the experience of target groups in the policy options (evidence generation). Incorporates a complex and dynamic view of change theory.	Presupposes a rational approach to policy-making. Not capture the diversity and complexity of policy problems and contexts. Neglects the unintended or unexpected effects of policy actions. Overlooks cognitive shortcuts and social norms in policy making. Assumes homogeneity between policy makers and researchers.
**TKPA**	New element of policy learning with data and information use (130). Scientific evidence and research results helped create accepted health plans (130). New component of policy learning better than Kingdon’s policy window (130). Action through agenda setting, coalition building and data and information learning (19).		Incorporates a complex and dynamic view of change theory.	Presupposes a rational approach to policy-making. Lacks alternatives for time-sensitive or crisis situations. Not capture the diversity and complexity of policy problems and contexts. Assumes availability and quality of evidence. Neglects the unintended or unexpected effects of policy actions. Overlooks cognitive shortcuts and social norms in policy making. Expects easy coalition and ignores divergent interests among stakeholders.
**KTA**	Clear and comprehensive framework for knowledge translation and IKT with social constructivism (133, 134). Guide for considering key elements in**knowledge translation** process (135). General and flexible implementation guide (134). Strong evidence base and validity (131).	No clear criteria for measuring success (138). No detailed guidance on action cycle steps. Unknown suitability as knowledge transfer account (77). Not applicable or relevant in low-income countries (119).	Adjusting knowledge creation and dissemination to user needs	Presupposes a rational approach to policy-making. Lacks alternatives for time-sensitive or crisis situations. Not capture the diversity and complexity of policy problems and contexts. Assumes availability and quality of evidence. Neglects the unintended or unexpected effects of policy actions. Overlooks cognitive shortcuts and social norms in policy making
	Systematic framework and methods for research translation, barrier overcoming, outcome measurement, and sustainability (24). Dynamic and interactive process for knowledge creation and improvement (136). Detailed picture of research use actions and strategies (137).	Lacking details on knowledge synthesis (137). Poor linkage between knowledge creation and action (137).		
**MLRA**	Detailed guidance on research dissemination (137). Accounting for research-policy factors (137).	Unclear dissemination/use factor (137). Design stage and other actors neglected (140). Unclear value/utilization of health information (141). Contextual challenges/opportunities ignored (141). Funding agencies’ priorities/responses unaddressed (142). Unrefined/unevaluated research evidence used (143).	Considers the general climate as the first step in linking research to action. Facilitates user pull with one-stop shop for high-quality reviews. Exploits teachable moments to profile high-quality reviews	Presupposes a rational approach to policy-making. Assumes availability and quality of evidence. Neglects the unintended or unexpected effects of policy actions. Overlooks cognitive shortcuts and social norms in policy making. Lacks guidance for dealing with ambiguous or contradictory evidence.
**SPIRIT**	Clear and practical guidance for studying causal pathways and testing effectiveness (144). Clear aim, sound knowledge base, practical guidance, and knowledge-building potential (106). Covers agenda-setting stage, diverse roles of research, and practical strategies and tools (19). Based on rigorous and systematic trial, refined and validated by evidence and feedback (145). Comprehensively and realistically depicts various influences on policy decisions (146).	Under evaluation, lacks published evidence, may need improvement, may face difficulties in being widely used (147). Ignores stakeholders’ role in EIPM and their evidence integration. Only focuses on research evidence use (148).	Considering catalyst: reason, context and timeliness of research use. Capacity assessment at the early stage.	Assumes availability and quality of evidence. Neglects the unintended or unexpected effects of policy actions. Unclear on how it uses and applies organizational change, and cognitive behavioral theory so overlooks cognitive shortcuts and social norms in policy making. Lacks guidance on addressing low capacity or selecting and tailoring strategies. Overlooks the tensions or conflicts between research rigor and policy relevance.

## Discussion

In this review, we examined nine models of EIPM in healthcare that have been proposed and applied in different contexts and settings. We identified some common strengths and limitations of these models based on existing critiques and our own analysis, which are summarized in Table 2. We found that these models have almost the same overall approach to the policy making. They are mostly from an academic perspective and, as Cairney and colleagues (153) stated, they offer three main strategies: enhancing the quality and relevance of evidence; providing information to policy makers; and facilitating collaboration and communication among stakeholders. These strategies are similar to those that have been suggested by recent scholars (39–41) who want to inject more evidence into the policy process. Interestingly, these strategies that are resembled in all nine models are also reminiscent of those that Weiss (154) discussed three decades ago. She explained that “the dominant approach to policy research over the years has assumed the existence of a benevolent and powerful decision maker.” She also listed various guidelines for researchers who want to influence policy makers, such as finding out who the key decision maker is; meeting with him or her personally; aligning the research with the questions he or she poses; engaging him or her in the research process; sharing results frequently and promptly; using simple language and concise summaries; ensuring that the results and the policy implications are realistic within the institutional context; and anticipating and preventing the potential difficulties in implementing the policy suggestions. It is surprising that the recommendations that Weiss mentioned, where she was already skeptical of these suggestions, have not changed much since then, even though a lot of scholarship has been done in this field (17).

The use of evidence in policymaking is both complex and dynamic, as the real world of policy making is complicated and chaotic. Policymakers routinely use a wide range of research and non-research evidence obtained from multiple actors and sources, which are subject to the challenges and contests of different networks with their own rules and frames. Policymakers also base their decisions on a combination of emotions, knowledge and shortcuts, which vary depending on the time, context and circumstance (155). Moreover, because policymaking processes are inherently political, so is their use of evidence, which may not match the normative expectations of how they should use research evidence (155). However, based on our analysis of the strengths and limitations of the models, we suggest that they can be grouped into three broad areas based on their underlying theoretical perspectives: sociology of knowledge, change theory and complexity science. We believe that considering concepts from these theoretical perspectives can help to form a more comprehensive and nuanced understanding of EIPM and to address these challenges.

Sociology of knowledge is one of the three broad areas that we suggest the strengths and limitations of the models can fall into. This area aims to understand how particular ideas, such as policy ideas or innovations, are constructed and promoted in the context of scientific activities. From this perspective, the quality of research may be less relevant to its potential influence than the way the ideas derived from research are received, translated and promoted (17). To illustrate this point, we will discuss one of the most influential approaches within sociology of knowledge: actor-network theory (ANT). According to ANT, policymaking and research are extremely complex processes, involving a diverse number of actors. It also suggests that the potential for major change is ever present, as the heterogeneous networks that underpin particular policy systems and ways of thinking might suddenly break down or reconfigure (17, 156). Moreover, ANT argues that ideas must be translated as they move between actors and across boundaries, which means that they are adapted and transformed in the process, rather than transferred unchanged like batons in a relay race (17). However, none of the models adequately account for the dynamics and diversity of the actors and networks involved in the production and dissemination of policy ideas or innovations. While some models acknowledge the importance of policy ideas or innovations, they do not specify how these ideas are modified or adapted as they cross different boundaries and contexts. They also do not address how external factors, such as values or power relations, shape the content and reception of these ideas.

Another theoretical perspective that can help us understand the strengths and limitations of the models is change theory, which is based on theories that explain how and why change occurs in social, political, economic or organizational systems (157, 158). Change theory recognizes that change is not a simple or rational process, but rather a complex and dynamic one that involves multiple factors and actors (157). Change theory also acknowledges that change is influenced by the historical and institutional context in which it takes place, and that past choices and events can constrain or enable the range of possible outcomes in the present and future. Concepts such as path dependency and historical institutionalism are useful for understanding how institutions shape and influence change processes and outcomes (157, 158). Although some of the models do consider the general climate of the organization that is the subject of change or innovation, such as its culture, values, norms, beliefs, etc., they do not offer sufficient solutions or strategies to deal with the challenges and opportunities that arise from these aspects. These aspects can also affect the receptivity and resistance to change or innovation within an organization. For example, some models do not provide guidance on how to deal with low organizational capacity, how to select and tailor strategies to suit different organizational contexts, or how to overcome institutional barriers or path dependencies that hinder change or innovation.

The last area that we will consider is complexity sciences, which provide a new way of thinking that challenges traditional mechanistic thinking and helps us understand the complex and difficult process of research use in policy making (159). Based on a constructivist approach, complexity sciences can accommodate various epistemological interests and offer methodological, conceptual and theoretical tools for studying research use, such as knowledge translation and implementation studies (159). Complexity theories can help us better understand and support EIPM by exploring and ‘capturing’ the diverse factors and processes involved in knowledge transfer, while cautioning us not to expect generalizable rules for increasing evidence uptake (160). One of these approaches is systems thinking: Systems thinking is a useful way to apply research and knowledge to health policy and practice (161). It looks at the whole system of elements, actors and context that affect how research is used. It helps to see how health issues and solutions are linked to many factors and actors, and how to adapt evidence and knowledge to different situations (161). However, none of the models that we reviewed in this article adequately capture the complexity and unpredictability of research use in policy and practice. They neglect the unintended or unexpected effects of policy idea or innovation that is offered, and they do not consider the complexity of the problem that the policy idea or innovation is supposed to address. Moreover, even though EIPP model (27) acknowledges that problems and solutions are not fixed or static, but dynamic and evolving, it does not explain the unintended consequences of the policy idea that is a potential policy action. Therefore, the models need to incorporate more complexity-based concepts and methods to better understand and support EIPM in healthcare.

Our work is not the first to review different models of evidence-policy interactions, but it adds to the existing literature by exploring the models and their features. We believe that this critical review can help to form a more comprehensive and nuanced understanding of EIPM and to address the challenges and opportunities that arise from the complexity and diversity of evidence-policy interrelationships. Several other articles have also reviewed different models of evidence-policy interactions, but with different scopes and focuses. For example, Votruba et al. (19) focused on mental health and LMICs; Milat and Li (24) focused on public health and health promotion; Baumann et al. (162) focused on dissemination frameworks; Tabak et al. (163) focused on dissemination and implementation frameworks; Strifler et al. (46) focused on knowledge translation theories, models and frameworks; and Davison et al. (164) focused on knowledge to action models or frameworks for promoting health equity. These articles also recognized the diversity and complexity of evidence-policy interrelationships, and the need for more comprehensive and nuanced frameworks to understand and facilitate research use. However, they did not critically explore different model of EIPM, as we did in our work.

## Limitation

This review has some limitations that should be acknowledged. First, we only searched three databases (PubMed, Scopus, Web of Science) for relevant papers, which may have resulted in missing some studies from other sources. These databases are comprehensive and widely used, so we believe that we captured the most pertinent frameworks and models for evidence-informed policy making. Another limitation is that we did not conduct a quality appraisal of the original articles that presented the models, as we justified in the methods section. We acknowledge that quality appraisal can be useful for some purposes and readers may have different opinions about the models. In addition, we excluded some models that were similar to previous ones or that only covered one aspect of the evidence-informed policy making process, as shown in Table 1. Furthermore, our strict inclusion criteria might have omitted some relevant studies that did not meet our definition of a framework or a model. Finally, our critique and analysis focused on application of the models at the legislative level of policymaking; their usefulness and limitations may differ when applied to administrative or clinical levels of policy.

## Conclusion

This study is the first critical review of different models of EIPM in healthcare, to our knowledge. We identified nine models that are applicable to healthcare decision making, and we described their strengths and limitations. Our aim was to provide a resource to facilitate the use of evidence in policy making by applying different models of EIPM. Besides our own critiques, we also considered other scholars’ opinions about the models. We acknowledge that the models have many strengths, but we also highlight their shortcomings in three broad areas: sociology of knowledge, change theory and complexity science. These shortcomings suggest that we need a model that can help us plan for different scenarios in policy making, taking into account the different environments and complexities of the problems. This study can be useful for decision makers who are interested in EIPM, as well as for researchers who want to explore different aspects of models in EIPM in healthcare.

## Author contributions

SJ: Conceptualization, Investigation¸ Methodology, Writing – original draft, Writing – review & editing. SY: Conceptualization, Methodology, Writing – review & editing. HP: Writing – review & editing. MM: Project administration, Supervision, Validation, Writing – review & editing.
