# Supplementary material for: From bench to policy: a critical analysis of models for evidence-informed policymaking in healthcare
Source: Front Public Health. 2024 Mar 26;12:1264315. doi: 10.3389/fpubh.2024.1264315 (PMC11002157; doi:10.3389/fpubh.2024.1264315)
Supplement: Supplementary file 1 [file Data_Sheet_1.docx]

**Supplementary material**

**Appendix 1: full search strategy with result**

**PubMed:
1461**

("Research dissemination"[Text Word] OR "research utilization"[Text Word] OR "research utilisation"[Text Word] OR "research to action"[Text Word] OR "research evidence"[Text Word] OR "evidence-based"[Text Word] OR "Evidence-Informed"[Text Word] OR "Knowledge Translation"[Text Word] OR "knowledge transfer"[Text Word] OR "knowledge exchange"[Text Word] OR "knowledge creation"[Text Word] OR "knowledge application"[Text Word] OR "Evidence utilization"[Text Word] OR "Evidence utilisation"[Text Word] OR "Evidence transfer"[Text Word] OR "knowledge generation"[Text Word] OR "research-informed"[Text Word] OR "knowledge utilization"[Text Word] OR "knowledge utilisation"[Text Word] OR "research transfer"[Text Word] OR "research uptake"[Text Word] OR "knowledge mobilization" [Text Word]) AND ("Health policy"[ MeSH Terms] OR "Decision-making"[Text Word] OR "Policy-makers"[Text Word] OR "Policy mak*"[Text Word] OR "Healthcare policy"[Text Word] OR "Policy making"[ MeSH Terms] OR "Decision mak*"[Text Word] OR "Policy decision*"[Text Word] OR "Policy development"[Text Word] OR "Policy Entrepreneur"[Text Word] OR "Public policy"[ MeSH Terms] ) AND ("Framework*"[Title] OR "Model*"[ Title] OR "Theoretical framework"[ Title] OR "Theor*"[ Title] OR "Conceptual framework"[ Title] OR "Conceptual model" [Title])

**Scopus:**

**2651**

( TITLE-ABS-KEY ( "Research dissemination" OR "research utilization" OR "research utilisation" OR "research evidence" OR "research to action" OR "evidence-based" OR "Evidence-Informed" OR "Knowledge Translation" OR "knowledge transfer" OR "knowledge exchange" OR "knowledge creation" OR "knowledge application" OR "Evidence utilization" OR "Evidence utilisation" OR "Evidence transfer" OR "knowledge generation" OR "research-informed" OR "knowledge utilization" OR "knowledge utilisation" OR "Research Use" OR "research-based knowledge" OR "research transfer" OR "research uptake" OR "knowledge mobilization" ) ) AND ( TITLE-ABS-KEY ( "Health policy" OR "Decision-making" OR "Decision-makers" OR "Policy-makers" OR "Policy making" OR "Healthcare policy" OR "Policy decision" OR "Policy development" OR "Public policy" ) ) AND ( TITLE ( "Framework" OR "Model" OR "Models" OR "Theoretical framework" OR "Theory" OR "Theories" OR "Conceptual framework" OR "Conceptual model" ) ) AND ( LIMIT-TO ( LANGUAGE , "English" ) )

**Web of Science:**

1508

(((TS=(“Research dissemination” OR ”research utilization” OR ”research utilisation” OR ”research evidence” OR ”research to action” OR ”evidence-based” OR ”Evidence-Informed” OR ”Knowledge Translation” OR ”knowledge transfer” OR ”knowledge exchange” OR ”knowledge creation” OR ”knowledge application” OR ”Evidence utilization” OR ”Evidence utilisation” OR ”Evidence transfer” OR ”knowledge generation” OR ”research-informed” OR ”knowledge utilization” OR ”knowledge utilisation” OR ”Research Use” OR ”research-based knowledge” OR ”research transfer” OR ”research uptake” OR ”knowledge mobilization”)) AND TS=(“Health policy” OR “Decision-making” OR “Decision-makers” OR ”Policy-makers” OR ”Policy making” OR ”Healthcare policy” OR ”Policy decision” OR ”Policy development” OR ”Public policy”)) AND TI=(“Framework” OR ”Model” OR ”Models” OR ”Theoretical framework” OR ”Theory” OR ”Theories” OR ”Conceptual framework” OR ”Conceptual model”)) AND (LA==("ENGLISH"))
